# Supplementary material for: Quantitative Phase Imaging of Spreading Fibroblasts Identifies the Role of Focal Adhesion Kinase in the Stabilization of the Cell Rear
Source: Biomolecules. 2020 Jul 22;10(8):1089. doi: 10.3390/biom10081089 (PMC7463699; doi:10.3390/biom10081089)
Supplement: Supplementary file 1 [file biomolecules-10-01089-s001.zip › supplementary correction/Supplementary figure legends.docx]

**Figure S1. Western blot analysis of FAK and Rack1 depletion.** The original results which have been used to create Figure 2A. The uncropped blots show expression levels of FAK and Rack1 proteins in control and siRNA treated cells. RSK was used as the loading control.

**Figure S2. Cell dry mass increase at the leading edge of Rat2 fibroblast correlates with GFP-LifeAct localization.** GFP-LifeAct localization and its superposition with quantitative phase images of a Rat2 fibroblast cell at the cell edge **(A)** and in presumed peripheral ruffles **(B)** are shown. The lower panels display a higher magnification of the boxed area. The graph shows dry mass distribution (magenta) and GFP-LifeAct intensity (green) in the imaged cell. The yellow arrowhead in (A) indicates the increase in dry mass and GFP-LifeAct signal at the leading edge. The yellow arrowhead in (B) indicates the increase in dry mass and GFP-LifeAct signal in peripheral ruffles. The cell border is indicated by the dashed line. Scale bars 10µm and 2.5µm (crop).

**Figure S3. FAK depletion in Rat2 cells.** Representative fluorescence microscopy images of control and FAK deficient cells stained 72 hours after siRNA transfection. Rat2 cells were transfected with FAK siRNA, replated on fibronectin coated coverslips, fixed and stained for FAK (green) and actin (red) to determine the extent of FAK depletion. Scale bars 20µm.

**Figure S4. Nucleus displacement to the cell periphery in a Rack1-depleted cell.** Greyscale or pseudo-coloured quantitative phase images of a Rack1 depleted cell are provided (insets represent the signal intensity scale). The graphs show the distribution of signal intensities (i.e. cell dry mass) of corresponding diagonal (blue) and cross (orange) sections. Cell borders are indicated by dashed lines. Scale bar 10µm.

**Figure S5. Actin and focal adhesions organization in Rat2 cells depleted of FAK, Rack1 or both FAK and Rack1.** Representative fluorescence microscopy images of Rat2 cells stained 72 hours after siRNA transfection. Rat2 cells were stained for FAK (green) and actin (red) to determine actin and focal adhesion organization. Two examples of FAK/Rack1 depleted cells are shown. Scale bars 20µm.

**Figure S6. The ends of FAK depleted cells display an increase in dry mass.** Greyscale or pseudo-coloured quantitative phase images of a continuously adherent cell depleted of FAK are provided (insets represent the signal intensity scale). The graphs show the distribution of signal intensities (i.e. cell dry mass) of corresponding diagonal (blue) and cross (orange) sections. Cell borders are indicated by dashed lines. The increase in dry mass at opposite cell protrusions is indicated by yellow arrowheads. Scale bar 10µm.

**Figure S7.** **Analysis of cell edge motility and convexity by QuimP software.** **(A)** The left panel shows two binary images from a time lapse video of a control cell, cell outlines are indicated in blue (frame 1) and red (frame 2). The middle panel is an overlay of the cell outlines obtained by QuimP software from a time lapse movie. Green circles indicate the sites where the cell outlines from two different time points intersect. The right panel shows the visualization of the ECMM tracking method (plugin for QuimP software) which chose randomly a point on the cell outline and assigned it the value zero (indicated as 0), other points are then calculated according to their distance from the “0” point. The length of the cell boundary is normalized to 1, the movement of points is shown using black lines. **(B)** The motility map represents the movement of every point from t0 to t0+1 point. Pixels are coloured according to the point speed, red colours represent contracting regions and blue colours expanding regions. The outlines of the cell at time frame t0 are shown in blue and at time frame t0+1 in red. Zero indicates the start point of the cell outline. **(C)** The convexity map represents the curvature of the cell boundary, measured in the range (-1,1), where negative values are concave (blue), and positive convex (red).

**Figure S8. Development of a round cell shape in spreading Rat2 fibroblasts depleted of Rack1. (A)** Series of pseudo-coloured quantitative images selected from time-lapse CCHM showing protruding and retracting areas in control Rat2 cells spreading on fibronectin. Time is indicated in h.min.s. **(B)** The time sequence of the cell outline during spreading of a Rack1 depleted cell superimposed from the first image to the last image. The first and last images of a spreading control cell are displayed, white arrows indicate the start point of the cell outline. **(C)** Cell edge motility and convexity maps of a spreading Rack1 depleted cell (see Fig. 3C for details). Scale bar 10µm.
