# Supplementary material for: Quantitative Phase Imaging of Spreading Fibroblasts Identifies the Role of Focal Adhesion Kinase in the Stabilization of the Cell Rear
Source: Biomolecules. 2020 Jul 22;10(8):1089. doi: 10.3390/biom10081089 (PMC7463699; doi:10.3390/biom10081089)
Supplement: Supplementary file 1 [file biomolecules-10-01089-s001.zip › supplementary correction/Supplementary Figure S7(final - checked).pdf]

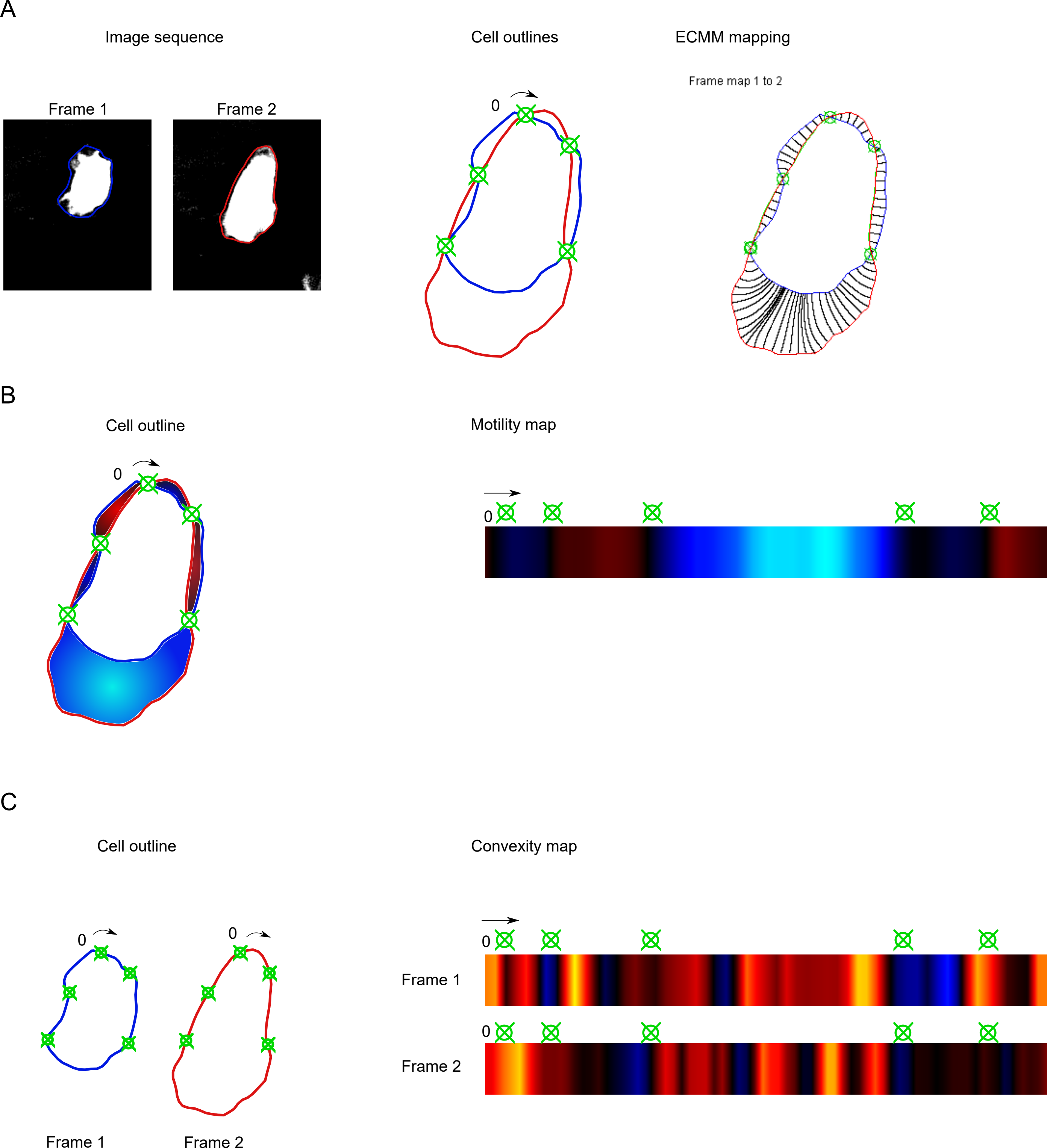
A
Image sequence
Frame 1
Frame 2
Cell outlines
0
ECMM mapping
Frame map 1 to 2
B
Cell outline
0
Motility map
0
C
Cell outline
0
0
Frame 1
Frame 2
Convexity map
0
Frame 1
0
Frame 2
