# Supplementary material for: Quantitative Phase Imaging of Spreading Fibroblasts Identifies the Role of Focal Adhesion Kinase in the Stabilization of the Cell Rear
Source: Biomolecules. 2020 Jul 22;10(8):1089. doi: 10.3390/biom10081089 (PMC7463699; doi:10.3390/biom10081089)
Supplement: Supplementary file 1 [file biomolecules-10-01089-s001.zip › supplementary correction/Video legends.docx]

**Video S1. Development of the cell rear and front in spreading Rat2 fibroblasts.** Cell spreading on fibronectin was followed for 2 hours with a capture frame of 1 minute. Left: CCHM QPI, middle: pseudocolored quantitative phase movie, right: cell contours from ECMM plugin (Quimp software) (not in scale). After 10 minutes of spreading there is visible formation of two distal protruding edges, which later (after 20 minutes) determine the cell front and rear and are separated by non-protruding cell regions.

**Video S2.** **Development of the elongated cell shape in a spreading Rat2 fibroblast depleted of FAK.** A spreading cell was followed for 2 hours with a capture frame of 1 minute. Left: CCHM QPI, middle: pseudocolored quantitative phase movie, right: cell contours from ECMM plugin (Quimp software) (not in scale). FAK depleted cells form two distally oriented continuously protruding regions that determine the prolonged bipolar shape of a FAK depleted cell.

**Video S3.The elongated cell phenotype of FAK depleted cells as a consequence of impaired tail retraction.** A spreading cell was followed for 5 hours with a capture frame of 1 minute. Left: CCHM QPI, middle: pseudocolored quantitative phase movie, right: cell contours from ECMM plugin (Quimp software) (not in scale). A FAK depleted cell is unable to retract the trailing edge resulting in cell elongation.

**Video S4. A RACK1 depleted cell adopts a round shape with symmetric protrusions along the cell outline.** The spreading of a RACK1 depleted cell on fibronectin was followed for 90minutes with a capture frame of 1 minute. Left: CCHM QPI, middle: pseudocolored quantitative phase movie, right: cell contours from ECMM plugin (Quimp software) (not in scale). During cell spreading, a RACK1 depleted cell is protruding in all directions.
