# Supplementary figures and images for: Quantitative Phase Imaging of Spreading Fibroblasts Identifies the Role of Focal Adhesion Kinase in the Stabilization of the Cell Rear
Source: Biomolecules. 2020 Jul 22;10(8):1089. doi: 10.3390/biom10081089 (PMC7463699; doi:10.3390/biom10081089)

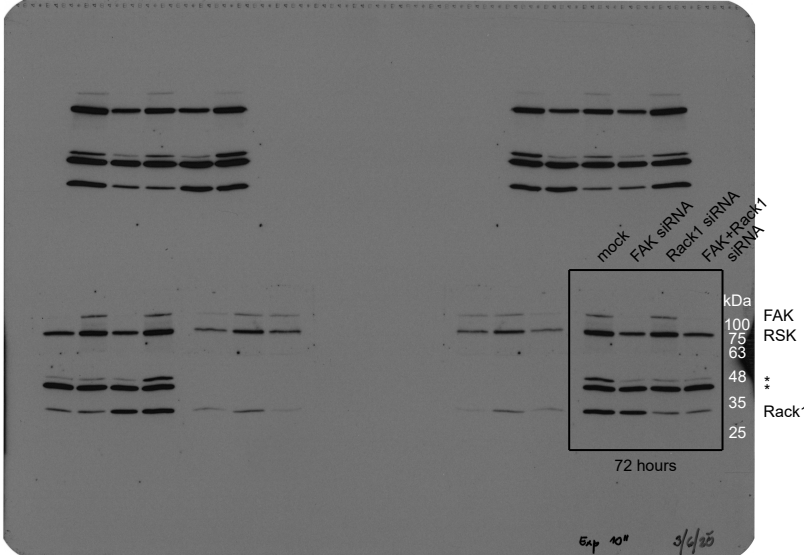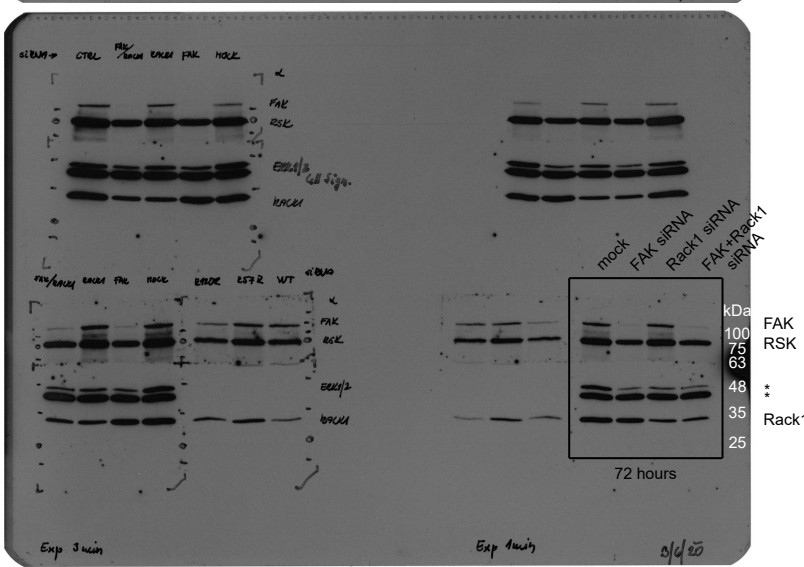

Supplement: Supplementary file 1 [file biomolecules-10-01089-s001.zip › supplementary correction/Supplementary Figure S1 (updated).pdf]

**A**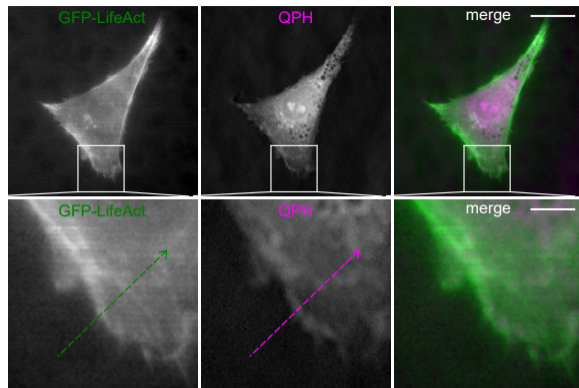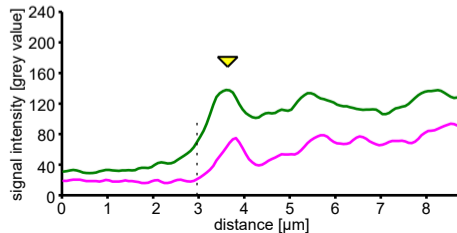**B**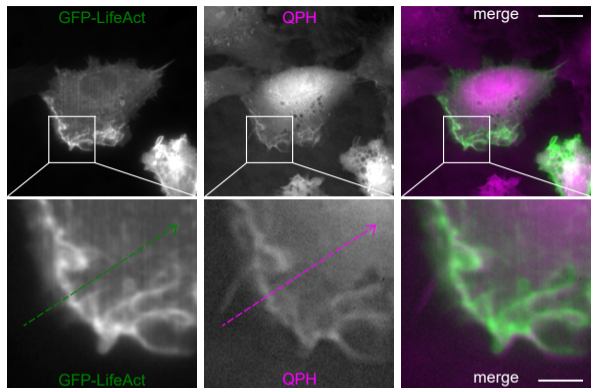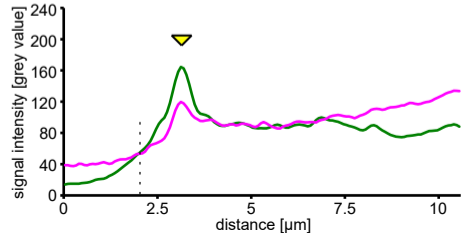

Supplement: Supplementary file 1 [file biomolecules-10-01089-s001.zip › supplementary correction/Supplementary Figure S2 (updated).pdf]

**FAK****Actin****Merge**

Ctrl

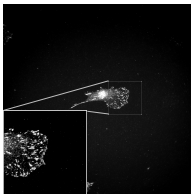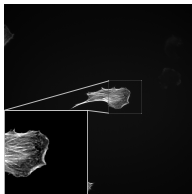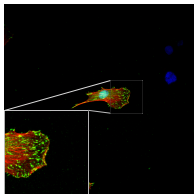

FAK KD

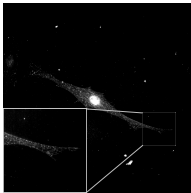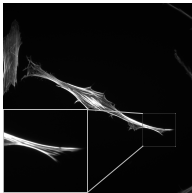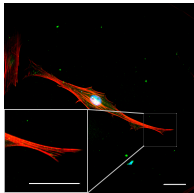

Supplement: Supplementary file 1 [file biomolecules-10-01089-s001.zip › supplementary correction/Supplementary Figure S3(final - checked).pdf]

Rack1 siRNA

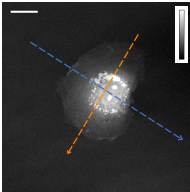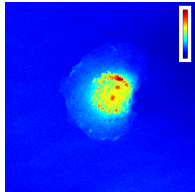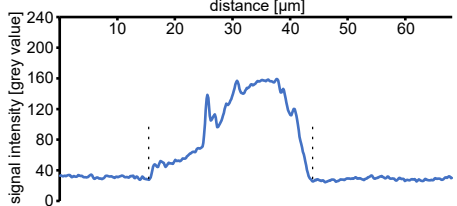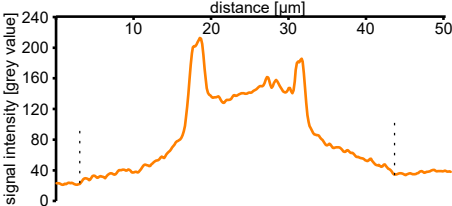

Supplement: Supplementary file 1 [file biomolecules-10-01089-s001.zip › supplementary correction/Supplementary Figure S4(updated).pdf]

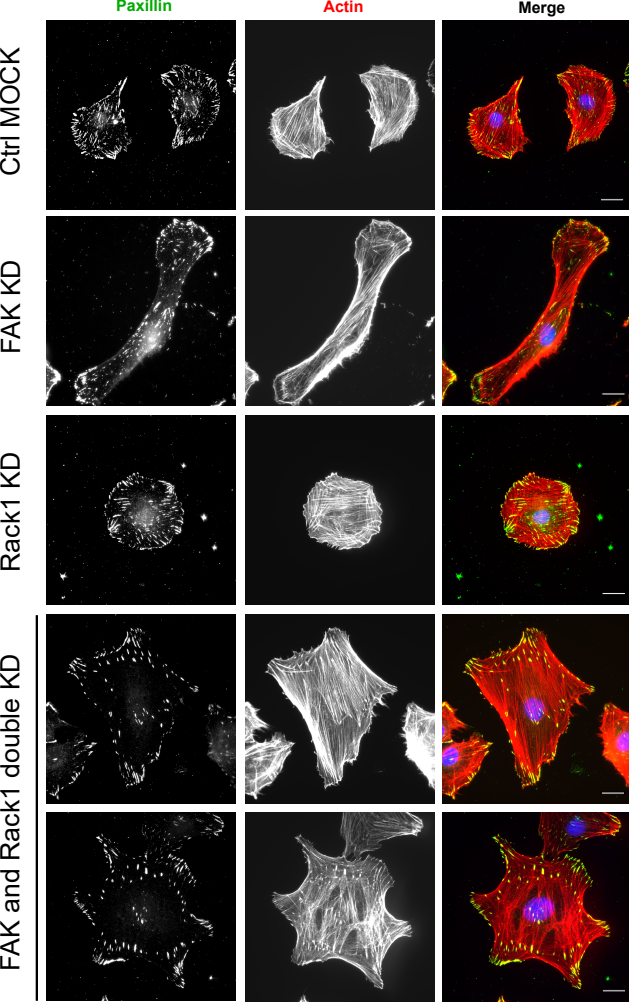

Supplement: Supplementary file 1 [file biomolecules-10-01089-s001.zip › supplementary correction/Supplementary Figure S5.pdf]

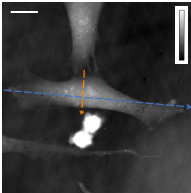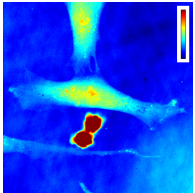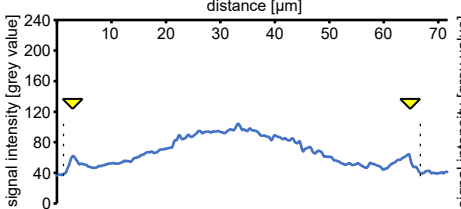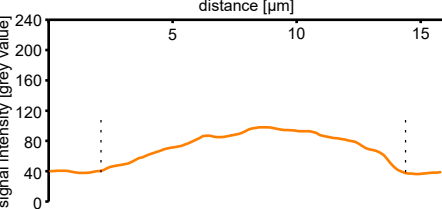

Supplement: Supplementary file 1 [file biomolecules-10-01089-s001.zip › supplementary correction/Supplementary Figure S6 (final - checked).pdf]

**A**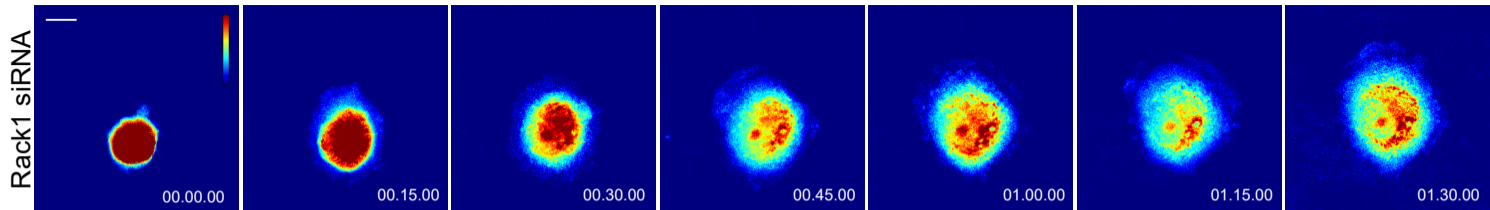**B**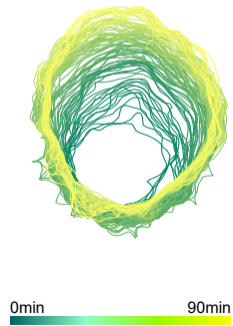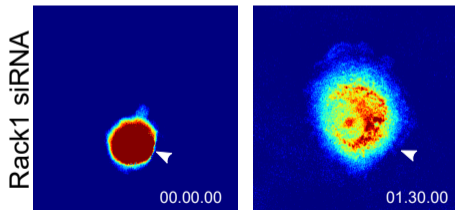**C**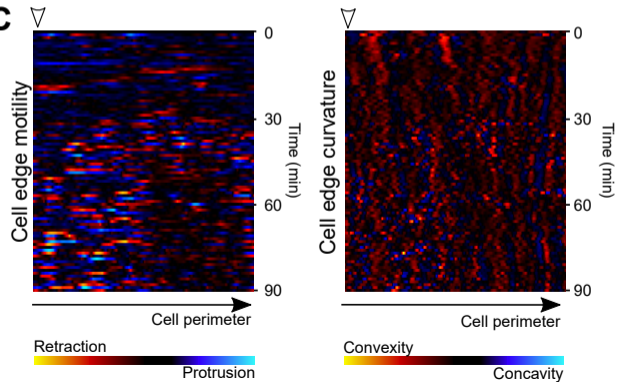

Supplement: Supplementary file 1 [file biomolecules-10-01089-s001.zip › supplementary correction/Supplementary Figure S8(updated).pdf]
